# Platelet-rich plasma (PRP) in dental and oral surgery: from the wound healing to bone regeneration

**DOI:** 10.1186/1742-4933-10-23

**Published:** 2013-06-13

**Authors:** Antonino Albanese, Maria E Licata, Bianca Polizzi, Giuseppina Campisi

**Affiliations:** 1 Department of Surgical, Oncological and Oral Sciences (Di.Chir. On.S.), Università degli studi di Palermo, Via del Vespro, 129, 90127 Palermo, Italy

**Keywords:** PRP, Wound healing, Bone regeneration, Dental surgery, Oral surgery, Tooth extraction, Periodontal surgery, Implant surgery, BRONJ

## Abstract

Platelet-rich plasma (PRP) is a new approach to tissue regeneration and it is becoming a valuable adjunct to promote healing in many procedures in dental and oral surgery, especially in aging patients. PRP derives from the centrifugation of the patient's own blood and it contains growth factors that influence wound healing, thereby playing an important role in tissue repairing mechanisms. The use of PRP in surgical practice could have beneficial outcomes, reducing bleeding and enhancing soft tissue healing and bone regeneration. Studies conducted on humans have yielded promising results regarding the application of PRP to many dental and oral surgical procedures (i.e. tooth extractions, periodontal surgery, implant surgery). The use of PRP has also been proposed in the management of bisphosphonate-related osteonecrosis of the jaw (BRONJ) with the aim of enhancing wound healing and bone maturation. The aims of this narrative review are: i) to describe the different uses of PRP in dental surgery (tooth extractions and periodontal surgery) and oral surgery (soft tissues and bone tissue surgery, implant surgery and BRONJ surgery); and ii) to discuss its efficacy, efficiency and risk/benefit ratio. This review suggests that the use of PRP in the alveolar socket after tooth extractions is certainly capable of improving soft tissue healing and positively influencing bone regeneration but the latter effect seems to decrease a few days after the extraction. PRP has produced better results in periodontal therapy in association with other materials than when it is used alone. Promising results have also been obtained in implant surgery, when PRP was used in isolation as a coating material. The combination of necrotic bone curettage and PRP application seem to be encouraging for the treatment of refractory BRONJ, as it has proven successful outcomes with minimal invasivity. Since PRP is free from potential risks for patients, not difficult to obtain and use, it can be employed as a valid adjunct in many procedures in oral and dental surgery. However, further RCTs are required to support this evidence.

## Introduction

Research in dental and oral surgery often involves materials and procedures which are capable of improving clinical outcomes in terms of percentages of success. The goal of this research was to find a treatment approach which could reduce bleeding, promote effective bone regeneration and rapid soft-tissue healing by employing resources which are easy to use at a modest cost.

Platelet rich plasma (PRP) is a new approach to tissue regeneration: it is widely used in various surgical fields, including head and neck surgery, otolaryngology, cardiovascular surgery, and maxillofacial surgery. Commonly, PRP is used in a gel formulation, which is formed by mixing PRP (derived from the centrifugation of autologous whole blood) with thrombin and calcium chloride. PRP gel includes a high concentration of platelets and a native concentration of fibrinogen [1,2].

During wound healing, platelets are among the first cells to respond at a wound site, being critical to the initiation of this process. Besides their procoagulant effects, platelets form a rich source of important growth factors, such as platelet-derived growth factor (PDGF), transforming growth factor-b (TGF-b) 1 and 2, and vascular endothelial growth factor (VEGF); all of these are involved in the angiogenic cascade which assists in hard and soft tissue wound healing [3-5].

Recently, PRP has become a valuable adjunct to promote healing in many procedures in dental and oral surgery. They include: ablative surgical procedures, mandibular reconstruction and surgical repair of the alveolar cleft, treatment of infrabony periodontal defects and periodontal plastic surgery, as well as procedures relating to the placement of osseointegrated implants. In such procedures, the adhesive nature of PRP facilitates the easier handling of graft material, with more predictable flap adaptation and hemostasis, and a more predictable seal than is the case with primary closure alone [6-14]. Recently, the use of PRP has also been proposed in the management of bisphosphonate-related osteonecrosis of the jaw (BRONJ) or avascular necrosis, which is caused by other factors (e.g. radio-osteonecrosis), with the aim of increasing wound healing and bone maturation [15-21].

Aging patients are usually the elective patients for these procedures. From a dental point of view, these patients could be considered as *special needs patient*, requiring a specific approach; age is considered an important determinant of periodontal disease, which is the main cause of tooth loss in adulthood. Moreover, elderly patients are mostly subject to systemic diseases which influence the response to surgical treatment in terms of coagulation and tissue repair. The improvement in quality of life of aging patients in recent decades has determined a growing request for elective treatments and devices which answer their specific needs contemporaneously [22,23].

The aims of this narrative review were: i) to describe the different uses of PRP in dental surgery and oral surgery; and ii) to discuss its efficacy, efficiency and risk/benefit ratio. The different bioactive substances present in PRP and the role played in the healing process will also be elucidated.

## Methods

As part of the review process, the literature was searched for published studies relating to PRP and dental surgery (e.g. alveolar wound healing and periodontal surgery) and oral surgery (i.e. soft tissues, bone tissues, and BRONJ surgery). A search of studies in the literature was conducted according to the Dickersin’s search strategy [24], using MeSH terms and text words. An extensive search of bibliographical databases included: MEDLINE (1966– November week 4, 2012); EMBASE (1988–November week 4, 2012); the Cochrane Library and Best Evidence (1991– third quarter 2012); the ISI Web of Science (1965– November week 4, 2012); PubMed (1966– November week 4, 2012); Lilacs (1982– November week 4, 2012); the Cochrane Central Register of Controlled Trials (1991–third quarter 2012) and CINAHL (1982– November week 4, 2012). The following keywords were used in searching for references: *PRP*, *Piastrinic Gel*, *PRF*, *Oral surgery*, *Dental surgery*, *Periodontal surgery*, *Oral medicine*, *Wound Healing*, *ONJ*, *BRONJ*. Other sources were taken from the reference list of selected Papers.

All RCTs and literature reviews were considered. Case reports were used for the BRONJ session only due to the absence of original articles relating to this topic. Articles published in English from 2007 to December 2012 were considered for the discussion of PRP efficacy and efficiency. Studies based on *in vitro* trials were excluded and a total of 68 articles were finally considered. The quality of RCTs was assessed, taking into consideration the degree of bias, the presence of randomization and blindness.

This Review is structured into three main sections: the first section deals with the action mechanism of PRP on tissue wound healing; the second describes the use of PRP in dental surgery (tooth extractions and periodontal surgery) and oral surgery (soft tissues and bone tissue surgery, implant surgery and BRONJ surgery) and its advantages in terms of efficacy and efficiency. The studies selected in this section are listed in Tables 1, 2, 3 and 4 for dental surgery and oral surgery respectively. A qualitative scale with three different scores (*weak*, *moderate and strong*) was used in the Tables to evaluate the PRP effect. The method of scoring the PRP effect as used in the Tables, is based on the statistical significance of the efficacy of PRP, as reported in the analyzed studies: a. weak: effective but not statistically significant; b. moderate: borderline efficacy, statistically significant; c. strong: efficacy statistically significant. The third and final section discusses the risk/benefit ratio associated with the use of PRP.

**Table 1:** Summary of the RCTs using PRP in tooth extraction

**Authors**	**Year of publication**	**Number of patients**	**Follow-up (wks)**	**Main results**	**Effect of PRP**
Alissa et al.	2010	23	12	Statistical significant improvement in soft and bone tissuehealing; statistically significant reduced post-operative pain and complications	strong
Ogundipe et al.	2011	11	12	Statistical significantly reduced pain; improvement in swelling/interincisal mouth opening and bone density but not statistically significant	moderate
Ruktowski et al.	2010	12	25	Early and significant increased radiographic density over baseline measurements in PRP- treated sites; no significant improvement in post-operative pain and bleeding after PRP application.	moderate
Celio-Mariano et al.	2012	15	1-4-8-12-24	Significant improvement in bone healing in PRP- treated sites	strong
Arenaz-Bua et al.	2012	82	12-24	No acceleration of bone formation after PRP treatment. No improvement in pain, swelling, trismus and infection.	weak
Gurbuzer et al.	2008	12	1-4	No increased osteoblastic activity in PRP treated sites	weak

**Table 2:** Summary of the RCTs using PRP in periodontal surgery

**Authors**	**Year of publication**	**Number of patients**	**Treatment**	**Follow-up (wks)**	**Main results**	**Effect of PRP**
Pradeep et al.	2009	20	Treatment of furcation defects	24	No complete closure of furcation defects	weak
Menezes et al.	2012	60	Treatment of infrabony defects	48-192	Positive effect of PRP used with other graft materials in infrabony defects but not when used alone	weak
Saini et al.	2011	20	Treatment of infrabony defects	12-24-36	Positive effect of PRP used with other graft materials in infrabony defects	moderate
Bharadwaj et al.	2011	10	Treatment of infrabony defects	24	Significant improvement in PD, CAL and bone radio-density	strong
Ozdemir et al.	2012	14	Treatment of infrabony defects	24	Positive effect of PRP used with other graft materials in infrabony defects but not when used alone	weak
Harnack et al.	2009	22	Treatment of infrabony defects	24	No improvement in PPD and CAL derived from the adjunt of PRP to other graft material	weak
Rodrigues et al.	2011		Treatment of infrabony defects	12-24-36	Better clinical results for PRP used with other graft materials in infrabony defects than with PRP used on its own	weak
Dori et al.	2008	26	Treatment of infrabony defects	48	No adjunctive benefit with the use of PRP	weak
Dori et al.	2009	30	Treatment of infrabony defects	48	No adjunctive benefit with the use of PRP	weak
Piemontese et al.	2008	60	Treatment of infrabony defects	48	No adjunctive benefit with the use of PRP	weak
Keceli et al.	2008	40	Root coverage	6-36-48	No adjunctive benefit with the use of PRP	weak

**Table 3:** Summary of the RCTs, using PRP in soft/bone tissue surgery and implant surgery

**Authors**	**Year of publication**	**Number of patients**	**Treatment**	**Follow-up (wks)**	**Main results**	**Effect of PRP**
Anitua et al.	2006	295	Implantology	8	Improvement in implant prognosis	strong
Anand et al.	2012	11	Implantology	12-24-36-48	Improved early bone apposition around the implant	strong
Gentile et al.	2010	15	Reconstructive surgery of the jaw	2-4-12-24	Efficacy of PRP treatment in terms of patient satisfaction and low-morbidity	strong
Wojtowicz et al.	2007	16	Augmentation of mandibular bone	12	PRP is more effective than bone marrow, containing CD34+ cells	strong
Daif	2012	24	Bone regeneration of mandibular fractures	1-12-24	Direct application of the PRP along the fracture lines may enhance bone regeneration in mandibular fractures	strong
Khairy et al.	2012	15	Sinus lift	12-24	PRP- enriched bone grafts were associated with superior bone density at 6 months post grafting	strong
Poeschl et al.	2012	14	Sinus lift	28	Increased new bone formation when PRP was used	strong
Cabbar et al.	2011	10	Sinus lift	28	No statistically significant differences were observed	weak

**Table 4:** Summary of the case reports using PRP in the BRONJ surgery

**Authors**	**Year of publication**	**Number of patients**	**Type of lesion**	**Treatment options**	**Follow-up (Mths)**	**Main results**	**Effect of PRP**
Curi et al.	2007	3	Jaw lesions		6-8	Resolution of all lesions	strong
Lee et al.	2007	2	Complications of dental implants: oral sinus communication and lesion on the jaw ramus	Closure of the oroantral communication by rotating a large palatally-based pedicle flap over the defect; surgical debridment of the lesion of the ramus	6-9	Resolution of pain and complete closure of exposed bone	strong
Adornato et al.	2007	12	Soft tissue ulcerations and bone exposure	Marginal resection limited to the alveolar bone	6	Ten patients showed complete soft tissue healing	strong
Cetiner et al.	2009	1	Exposed necrotic bone in the alveolus	Marginal resection of the mandibular necrotic bone	6	Complete healing of the oral mucosa and alveolar bone at the surgical site	strong
Bocanegra et al.	2012	8	Exposed necrotic bone in the mandibula and maxilla	Removal of necrotic bone and curettage of the underlying bone	14	Fast mucosal healing, reduced need for analgesics and resolution of mouth lesions, without evidence of exposed bone.	strong
Mozzati et al.	2012	32	Jaw lesions	Resection of the necrotic bone		The orthopanoramic X-ray and computed tomography performed before and after surgery showed successful outcomes	strong
Coviello et al.	2012	7	Jaw lesions	Surgical debridement and sequestrectomy	3	Improvement in wound healing and reduction of bone exposure	strong

### The PRP action mechanism

Platelet-rich plasma (PRP) is defined as a high concentration of autologous platelets in a small volume of autologous plasma [25,26]. Specifically, PRP is a platelet concentration with at least 1,000,000/1 L in a 5 mL volume of plasma, when normal human platelet counts in the blood range from 150,000/1 L to 350,000/1 L. The platelets contained in this concentrate of autologous plasma release their alpha granules after the coagulation process has been locally trigged in the wound site. These alpha granules contain a cocktail of growth factors which promote proliferation, chemotaxis and the differentiation of cells, which are essential to osteogenesis. Thus, besides its procoagulant effect, PRP is a source of growth factors involved in initiating and sustaining wound healing by accelerating bone repair, promoting fibroblast proliferation, and increasing tissue vascularity [27].

Platelet-rich plasma gel is formed by mixing PRP (derived from the centrifugation of autologous whole blood) with thrombin and calcium chloride. Adding thrombin and calcium chloride to PRP automatically activates the alpha granules to release the following biological growth factors: platelet-derived growth factor (PDGF), transforming growth factor-beta (TGF-b), vascular endothelial growth factor (VEGF), insulin- like growth factor I, epidermal growth factor (EGF) and epithelial cell growth factor [3].

The major effects of PRP are derived from PDGF, which has been identified as an important protein for hard- and soft-tissue healing. PDGF has been shown to stimulate chemotaxis, mitogenesis and the replication of stem cells at the site of a wound to the site of tissue injury. This results in the formation of matrix bone and angiogenesis by stimulating increased levels of VEGF. This in turn may lead to accelerate soft-tissue healing due to neo-vascularization. PDGF also stimulates the production of fibronectin, a cell adhesion molecule used in cellular proliferation and migration during healing, including osteoconduction and hyaluronic acid, and it assists in promoting wound contraction and remodelling [28].

Other cytokines released by PRP alfa granules are TGF-b1 and TGF-b2, both of which are involved in connective tissue repair and bone regeneration. Their most important role appears to be to stimulate fibroblast chemotaxis and the production of collagen and fibronectin by cells while inhibiting collagen degradation by decreasing proteases and increasing protease inhibitors. *In vitro* and *in vivo* studies have also shown that TGF increases the proliferation of mesenchymal stem cells and osteoblasts, leading to bone regeneration. Specifically, TGF-b2 has been shown to increase osteoblast and osteoclast activity. An increase in TGF-b2 may accelerate bone regeneration by controlling the activity of osteoblasts and osteoclasts [29,30].

### The use of PRP in dental surgery

#### The effects of PRP on healing the alveolar socket after tooth extraction

Tooth extraction is a common dental procedure which involves severely decayed, periodontally affected, not-restorable or impacted teeth. These procedures can be associated with significant postoperative pain, particularly when third impacted molars are extracted. Furthermore, prolonged bleeding can be experienced by patients especially by those undergoing anticoagulant therapy [31]. To address post-operative discomfort and to enhance tissue repair mechanisms, many procedures (i.e. fibrin sponge, biostimulation with LASER) have been performed which promote the healing process [32,33].

Recently, the use of PRP has been proposed as a way of obtaining high concentrations of growth factors involved in tissue healing and regeneration. The therapeutic strategy of this approach is to promote the process of tissue repair, improving the quality of healing and healing time [8]. However, very few studies have been carried out on humans and contradictory results have been produced regarding the efficacy of PRP. Promising results were reported by Alissa *et al*. (2010), who conducted a pilot study on the effect of PRP on the healing of the hard and soft tissues of extraction sockets. Soft tissue healing was significantly improved in patients treated with PRP compared with patients of the control group (no treatment). Moreover, patients untreated with PRP experienced complications (dry sockets and acutely inflamed alveolus), which were considered to be borderline statistically significant. Radiographic evaluation revealed a statistically significant difference only for sockets with a dense homogeneous trabecular pattern. Of interest, Alissa *et al*. (2010) also analyzed the post-operative pain of patients of the two groups (treated and untreated) and they reported significantly more pain in the control group, especially in the first three days post intervention [34].

A significant response to pain in patients undergoing a surgical extraction of a single impacted third molar and using PRP was also reported by Ogundipe *et al*. (2011). Moreover, an improvement in swelling and the interincisal mouth opening was obtained in these patients: the scores for lamina dura, trabecular pattern, and bone density were much improved among patients in the PRP group, even if this difference was not statistically significant [35].

Similar findings have been reported by Ruktowski *et al*. (2010) who used digital radiography and Computer Tomography (CT) scan analysis to track changes in radiographic density at PRP- treated sites in comparison to ipsilateral not-PRP treated sites. The PRP- treated sites demonstrated early and a significant increased radiographic density over baseline measurements following tooth removal. The greatest benefit attributed to PRP is during the initial 2-week post-operative healing time period: 6 weeks for control extraction sites to reach comparable bone density were required whereas PRP- treated sites achieved this at week 1. Post-operative pain and bleeding were not significantly affected by PRP application. [36]. Likewise, a more recent study by Celio-Mariano *et al*. (2012) showed a greater radiographic bone density in the PRP group, thereby demonstrating a significant improvement in bone healing in the sockets after extraction of mandibular third molars as compared to the control group [37].

In a prospective split-mouth study conducted by Arenaz-Bua *et al*. (2010) the efficacy of PRP in promoting bone regeneration after third molar extraction was analyzed. The Authors observed no further acceleration in bone formation at 6 months nor did they observe statistically significant differences between the groups regarding pain, swelling, trismus and infection throughout the post-operative period. [38]. Similarly, in a study by Gurbuzer *et al*. (2008) (using scintigraphy), the application of PRP on its own to soft tissue impacted mandibular third molar extraction sockets failed to increase the osteoblastic activity in post-surgical weeks 1 and 4 in comparison to non-PRP-treated sockets [39].

The above review of the literature suggests that the use of PRP in the alveolar socket after tooth extractions is certainly capable of improve soft tissue healing but there is insufficient evidence which supports the efficacy of PRP in improving bone regeneration. Similarly, the efficiency of PRP is controversial since the use of PRP in tooth extraction sites seems to influence the early phase of bone healing, thereby facilitating and accelerating bone formation in the initial period after tooth extraction, its influence decreasing after a few days. Not univocal results were also obtained for post-operative pain but conclusive considerations in terms of efficacy and efficiency could not be formulated. The aforementioned studies are listed in Table 1.

#### The use of PRP in periodontal surgery

The growth factors present in PRP are capable of forming a fibrin clot, promoting fibroblast proliferation and up-regulating collagen synthesis in the extracellular matrix. Thus, the use of PRP at injury sites might be able to promote wound healing and the regeneration of periodontal soft tissues [36]. Moreover, the ability of these factors to accelerate bone repair by increasing the mitosis of osteoblasts and tissue vascularity might be useful in the treatment of infra-bony defects [40,41]. However, the therapeutic efficacy of PRP in periodontal therapy still remains controversial.

The results of a recent systematic review regarding the efficacy of PRP in periodontal therapy have revealed that it is capable of improving gingival recession but not clinical attachment level in chronic periodontitis [42]. Moreover, Pradeep *et al*. (2009), who conducted a study on the treatment of mandibular furcation defects, have reported the lack of complete closure of furcation defects despite a significant improvement; this implies a limited role for autologous PRP as a regenerative material [43]. However, the efficacy of PRP on its own is difficult to evaluate since the majority of studies have been conducted by testing PRP in combination with graft materials in order to enhance the outcome of regenerative surgery. Moreover, a barrier membrane was used to cover the defects in most clinical studies and, thus, the effects of PRP may have been masked by the effects of the barrier.

The results of the systematic review by Del Fabbro *et al*. (2011) revealed that PRP may exert a positive adjunctive effect when used in combination with graft materials for the treatment of intrabony defects. However, no significant benefit of PRP was found for the treatment of gingival recession [44]. Similarly, two controlled clinical trials investigating the efficacy of PRP combined with other graft materials in the treatment of intraosseous periodontal defects reported a significantly more favorable clinical improvement in periodontal sites treated with the combination of PRP and the graft material than in those treated with the graft material alone [45,46]. Contemporaneously, Bharadwaj *et al*. (2011) found that the adjunct of PRP to bone graft appeared to be beneficial in the treatment of human periodontal intrabony defects. [47].

Different results have been reported by other Authors, who showed no significant benefits regarding the additional use of PRP to graft materials in the treatment of infra-bony defects. Ozdemir *et al*. (2012) showed that PRP combined with a graft material was effective in the treatment of intrabony defects after a 6-month healing period but no additional statistically significant improvements were observed when PRP was used [48]. Similar results were reached by Harnack *et al*. (2009) using the same combination of materials [49]. Rodrigues *et al*. (2011) concluded that both PRP and PRP combined with bovine anorganic bone mineral (ABM) resulted in a significant clinical improvement for the treatment of human periodontal intrabony defects but there was a preponderance of improved clinical results with the addition of ABM to PRP [50]. No additional effects were found by Döri *et al*. (2008, 2009) and Piemontese *et al*. (2008). Throughout all their studies, they concluded that the use of PRP failed to enhance the results obtained with the use of the graft material used on its own [51-53].

Few clinical comparative studies have investigated the use of PRP in the treatment of gingival recession. Keceli *et al*. (2008) did not reveal promising results and nor did they observe differences in clinical outcomes between connective tissue grafts (CTG) and a CTG-PRP combination [54]. The results of this analysis reflect the limited and heterogeneous data available and suggest that the specific selection of agents/procedures combined with PRP could be important. The aforementioned studies are listed in the Table 2.

### The employ of PRP in oral surgery

#### The use of PRP in soft tissues and bone tissues surgery and implant surgery

Animal and human studies have demonstrated that PRP enhances and accelerates soft tissue repair and bone regeneration [3,55]. In the field of bone tissue surgery, a recent study by Daif (2012) investigated the effect of autologous PRP on bone regeneration in mandibular fractures. He concluded that direct application of the PRP along the fracture lines may enhance bone regeneration [56]. Wojtowicz *et al*. (2007) compared the effects of stimulating the osteogenesis of the alveolar bone by transplants of autologous bone marrow and freshly-isolated mononuclear cells from bone marrow, containing CD34+ cells and PRP. It was shown that newly- formed bone increased under the influence of PRP. This treatment was more effective than that using the population of CD-34 bone marrow- derived stem cells [57].

A Cochrane review of Esposito *et al*. (2010) concluded that PRP treatment did not seem to improve the clinical outcome of sinus lift procedures with autogenous bone or bone substitutes [58]. In addition, a study by Khairy *et al*. (2012) evaluated the bone quality in sinus which had been augmented with autogenous bone with or without PRP. The conclusion was that enrichment with PRP did not significantly improve bone density at 3 months post grafting but PRP- enriched bone grafts were associated with superior bone density at 6 months post grafting [59]. Poeschl (2012) obtained successful results when PRP was used in combination with a graft material in maxillary sinus augmentation [60]. Cabbar (2011) *et al*. compared a bovine bone xenograft with PRP and without PRP to augment the human maxillary sinus in preparation for receiving dental implants. The conclusion of this study stated was that the combination of the xenograft and PRP did not have any effect on new bone formation and implant stabilization [61].

The preparation of PRP, as applied to an implant surface, adheres to metal and might create a new dynamic surface which could potentially show biological activity. In 2006, Anitua showed that the osseointegration of implants was enhanced by coating the implant surface with PRP prior to insertion into the alveolus [62]. Similarly, Gentile *et al*. (2010) reported their experience on 15 cases, including reconstructive surgery of the jaw, post-extraction alveolar bone regeneration, and oral implantology. The results of their study revealed the efficacy of the PRP treatment in terms of post-operative patients' satisfaction and low-morbidity [63]. Anand *et al*. (2012) have recently proposed that the use of a novel technique (of coating the implant with PRP) could improve the prognosis of the treatment regarding an immediate loading protocol [64].

The results of these studies demonstrate that PRP is effective in soft tissue healing and bone regeneration. The combination of PRP application with other biomaterials seems to be promising as regards sinus lifting, but the results depend on the material used. Promising results have also been obtained in implant surgery, using PRP on its own as a coating material. The aforementioned studies are listed in Table 3.

#### The use of PRP in BRONJ surgery

Some researchers have proposed the use of PRP in BRONJ surgery [15-21]. BRONJ is currently recognized as a significant complication, which is related to the use of bisphosphonates (BPs), a widely-used class of drugs employed in the preventative treatment of various pathologies joined by the same alteration of bone turn-over (i.e. osteoporosis, bone metastasis, associated with solid tumours and multiple myeloma, malignant hypercalcemia). BPs are capable of inhibiting osteoclast-mediated bone resorption, also displaying anti-angiogenetic activity. The bones of patients treated with BPs are, therefore, poorly vascularized and poorly supplied with the substances necessary for wound healing [65,66].

The use of these drugs could delay the onset of BRONJ, which is defined as an avascular area of necrotic bone in the maxillofacial area, with or without exposed bone. This area of osteonecrosis always appears in the traumatized bone. Although some of the cases reported were asymptomatic, most of them resulted in complications, such as an altered sensation in the affected area (e.g. mandibular alveolar nerve), purulent exudates, oral-cutaneos fistula, and mandibular fractures [65]. BRONJ management is currently controversial, ranging from medical to surgical treatment, with no definitive standard of care. Indeed, the response to radical surgery is less predictable than in other situations involving bone necrosis, such as radiotherapy or osteomyelitis [67]. Aggressive surgical debridement is also controversial due to the risk of worsening bone exposure. Occasionally, the bone is left exposed due to the difficulty of treating the lesion [68].

PRP therapy has been proposed as a complement to conservative surgery in order to enhance bone healing. The rationale for the employment of PRP in patients affected by BRONJ is based on the thesis that the presence of growth factors (usually repressed by BPs) constitutes a substitute stimulation to bone healing, which is similar to physiological healing. The growth factors in PRP might accelerate epithelial wound healing, decrease tissue inflammation after surgery, improve the regeneration of bone and soft tissues, and promote tissue vascularization. The additional advantages related to the use of this product are its biocompatibility and safety, as an autologous product [1,3].

Few case series studies relating to use of PRP in treating BRONJ have been published: Cetiner *et al*., 2009 described a case of zoledronate-associated BRONJ after tooth exodontia in a 68-year-old man with multiple myeloma, which was treated with surgical debridement plus PRP with a positive outcome after a 6-month follow-up period [15]. Curi *et al*., 2007 reported using this treatment in three cases of jaw lesions, which were followed up for 6 months in two cases and 8 months in one [16]. Lee *et al*., 2007 reported two cases which were successfully treated, which were secondary to complications of dental implants: one involved left oral sinus communication (with a 9-month follow- up period) and the other case involved a lesion on the left jaw ramus (6-month follow-up) [17].

In the study conducted by Bocanegra *et al*. (2012) eight patients were selected and all improved over a mean period of 3 weeks (2–4 weeks) after treatment with: fast mucosal healing, a reduced need for analgesics and a resolution of mouth lesions. These patients continued with follow-up visits, without any evidence of exposed bone after 14 months [18]. Adornato *et al*. (2007) treated twelve patients who presented with soft tissue ulcerations and bone exposure with measurements ranging from 5 to 25 mm. These lesions had not responded to six months of treatment with cleaning therapies, 0.12% chlorhexidine rinses and intermittent antibiotic therapies. These patients were treated with conservative marginal resections of the alveolar bone with primary closure over the bony defect, PRP and a resorbable membrane under antibiotic coverage. After six months, ten patients showed complete soft tissue healing with one patient displaying a recurrence of epithelial dehiscence; another patient, with recovery by secondary intention, did not show any regression of bone exposure [19].

Positive results were also obtained by Mozzati *et al*. (2012), who conducted a study on 32 patients treated with intravenous BPs for oncological pathologies affected by BRONJ. The patients were treated by a resection of the necrotic bone with primary closure of the mucosa over the bony defect using PRP. The orthopanoramic and computed tomography performed before and after surgery revealed successful outcomes [20]. Similarly Coviello *et al*. (2012), who reported the cases of 7 patients with multiple myeloma treated with BPs, concluded that the use of PRP enhances wound healing and reduce bone exposure and would be an effective treatment protocol to use in BRONJ subjects [21]. The results of these studies showed that the combination of necrotic bone curettage and PRP application seem to be promising for the treatment of refractory BRONJ. Since an efficient standard treatment has not yet been established, this combined approach can be considered a treatment option as it has demonstrated successful outcomes and minimal invasivity.

### The risk/benefit ratio and the use of PRP

PRP is an autologous preparation, utilizing the patient’s own blood in a significantly small quantity. For this reason, it is safe and there have been no published references relating to the risk of infections, disease transmission (such as HIV, hepatitis, or Creutzfeldt-Jacob disease), immunogenic reactions or any other adverse effects which exist with allografts or xenografts [69].

In the past, the use of bovine thrombin (an activator which allows polymerization of fibrin into an insoluble gel), used in the preparation of PRP, was associated with the risk of life-threatening coagulopathies [42]. However, the adverse reactions reported were related to the source and quantity of thrombin used. The use of bovine thrombin in PRP in low doses (<200 units), topically with no entry into systemic circulation and already clotted when coming into contact with human tissues, would not be dangerous as an immunologic reaction. [3]. Moreover, in the second generation platelet concentrate, PRP activation was effected using only calcium chloride, thus eliminating the risk associated with thrombin. No adverse effects linking the use of calcium chloride have been reported in the literature [70,71].

Although no undesirable effects have been reported in the many clinical cases subjected to PRP therapy, hypotheses as to the over-expression of growth factors and their receptors related to tumour and dysplastic tissues have been postulated. These hypotheses are founded on the fact that growth factors appear to regulate different cellular processes, such as mitogenesis, chemotaxis, cell differentiation and metabolism. However, the phenomenon leading to neoplastic growth requires more continuous doses of growth factors over time than those applied in PRP therapy and sufficient delivery, taking into account that extracellular growth factors degrade within 7–10 days. Moreover, there are previously existing alterations for developing a neoplasm and, in any case, the use of PRP should be avoided: in patients with precancerous oral conditions and in the vicinity of precancerous lesions (oral leukoplakia, erythroplasia or solar cheilitis); areas of oral epithelial dysplasia; and in patients with a prior history of exposure to carcinogens or primary oral squamous cell carcinoma [72].

The only disadvantage of PRP preparations would be the cost versus the outcome benefit. The doubtful success of PRP may not justify the cost to the clinician of buying the PRP-processing system and the disposable kits or the cost to the patient for paying for this treatment. Furthermore, an additional but less important inconvenience for the treatment would be that patients have to be subjected to a venipuncture and blood drawing procedure in preparation for PRP [1,3]. On the other hand, PRP has the advantage to being easily obtainable and not time-consuming for the patient and/or clinician. Even if preparation of PRP involves an additional step to the surgical procedure, it takes approximately 30 minutes and is best performed by a surgical assistant under the supervision of a trained dental surgeon. This can be done simultaneously while performing the surgery, and it, therefore, does not significantly increase the chair time of the operator and the patient [3,47].

## Conclusion

PRP preparations have been proposed for several uses in dental and oral surgery. The ease with which these preparations are used might be helpful to the dental professional in many surgical procedures, and their safety might encourage their wide employment [1,3,47,69]. The scientific evidence regarding the efficacy and efficiency of PRP is still controversial, given the paucity of RCTs relating to this topic and, of these, the majority has been conducted using different graft materials and applying different procedures.

This Review of the literature suggests that the use of PRP in the alveolar socket after tooth extractions is certainly able to improve soft tissue healing and positively influence bone regeneration but this latter effect seems to decrease a few days after extraction. PRP has revealed better results in periodontal therapy in association with other materials than when it is used alone, suggesting that the specific selection of agents/procedures combined with PRP could be important [34-39].

Promising results have also been obtained in implant surgery, using PRP on its own as a coating material. Furthermore, the combination of PRP application with other biomaterials seems to be favorable as regards sinus lifting even if the choice of material used is critical in this field [58-64]. The combination of necrotic bone curettage and PRP application seem to be encouraging for the treatment of refractory BRONJ, as it has demonstrated successful outcomes with minimal invasivity [15-21]. Since it is free from potential risks to patients, not difficult to obtain and use, PRP can be employed as a valid adjunct to many procedures in oral and dental surgery. However, further RCTs are required to support the use of PRP in current practice.

## Competing interests

The authors declare the absence of any competing financial interest in the writing of this review.
